# Characterization of the Immune Cell Infiltration Landscape in Esophageal Squamous Cell Carcinoma

**DOI:** 10.3389/fonc.2022.879326

**Published:** 2022-07-07

**Authors:** Zhilin Sui, Xianxian Wu, Longde Du, Han Wang, Lijuan Yuan, Jian V. Zhang, Zhentao Yu

**Affiliations:** ^1^ Departments of Esophageal Cancer, Tianjin Medical University Cancer Institute and Hospital, National Clinical Research Center for Cancer, Tianjin’s Clinical Research Center for Cancer, Key Laboratory of Cancer Prevention and Therapy, Tianjin, China; ^2^ Center for Energy Metabolism and Reproduction, Shenzhen Institute of Advanced Technology，Chinese Academy of Sciences; Shenzhen Institute of Advanced Technology, Chinese Academy of Sciences; Shenzhen Key Laboratory of Metabolic Health, Shenzhen, China; ^3^ Department of Thoracic, National Cancer Center/National Clinical Research Center for Cancer/Cancer Hospital & Shenzhen Hospital, Chinese Academy of Medical Sciences and Peking Union Medical College, Shenzhen, China; ^4^ Department of Pathology, National Cancer Center/National Clinical Research Center for Cancer/Cancer Hospital & Shenzhen Hospital, Chinese Academy of Medical Sciences and Peking Union Medical College, Shenzhen, China

**Keywords:** oesophageal squamous cell carcinoma, tumour microenvironment, immunotherapy, immune signatures, prognosis

## Abstract

**Background:**

Immunotherapy has achieved remarkable efficacy in treating oesophageal squamous cell carcinoma (ESCC). However, this treatment has limited efficacy in some patients. An increasing number of evidence suggested that immune cells within the tumour microenvironment (TME) are strongly related to immunotherapy response and patient prognosis. Thus, the landscape of immune cell infiltration (ICI) in ESCC needs to be mapped.

**Methods:**

In the study, the ICI pattern in 206 cases of ESCC was characterised by two algorithms, namely, CIBERSORT and single-sample gene set enrichment analysis (ssGSEA). The ICI score of each specimen was calculated by principal component analysis (PCA) according to ICI signature genes A (ICISGA) and B (ICISGB). The prognostic difference was evaluated by using the Kaplan–Meier method. The related pathways of ICI score were investigated by applying gene set enrichment analysis (GSEA). The R packages of ‘regplot’, ‘timeROC’ and ‘rms’ were applied for the construction of nomogram model.

**Result:**

Three TME subtypes were identified with no prognostic implication. A total of 333 differentially expressed genes (DEGs) among immune subtypes were determined, among which ICISGA and ICISGB were identified. Finally, ICI scores were constructed, and the patients were grouped into high or low ICI score group. Compared with the low ICI score group, the high ICI score group had better prognosis. GSEA revealed that the high ICI score group referred to multiple signalling pathways, including B cell receptor, Fc gamma R-mediated phagocytosis, NOD-like receptor and TGF-β signalling pathways. In addition, the nomogram model was constructed to evaluate 1-, 3- and 5-year probability of death in an ESCC patient. The ROC and calibration curves indicated that the model has a good discrimination ability.

**Conclusion:**

We depicted a comprehensive ICI landscape in ESCC. ICI score may be used as a predictor of survival rate, which may be helpful for guiding immunotherapy in the future.

## Introduction

Oesophageal carcinoma (EC) is among the most common gastrointestinal malignancies with approximately 572,034 new cases yearly (1). Oesophageal squamous cell carcinoma (ESCC) is the main subtype of EC and accounts for appropriately 90% of EC cases worldwide (2, 3). Despite advancements in multidisciplinary therapeutic approaches, the prognosis of ESCC remains unsatisfactory (4). Immune checkpoint inhibitors targeting PD1/PD-L1 have clinical efficacy in multiple cancers, including ESCC (5–7). Immunotherapy stimulates the patients’ immune response against malignant cells by targeting the immune checkpoint pathway (8). Despite the survival benefits of immunotherapy, only a small percentage of patients with ESCC (14%–28%) are benefitted (9–11). Therefore, effective biomarkers to guide patient selection and determine combination therapies are urgently needed.

Increasing evidence has elucidated the importance of the tumour microenvironment (TME), which is composed of a variety of cancer cells, infiltrating immune cells and stromal cells (12, 13). The heterogeneity of the TME, including immune-promoting cells, immunosuppressive cells and immune-related pathways, have been reported in patients with cancer (14–16). Changes in the number or functional activation of immune cells in the TME affect patient survival and response to immunotherapy in malignancies (17, 18). A growing number of studies have shown that intercellular interaction is more important than single cell subsets for anti-tumour effects. For example, exhausted T and NK cells are reportedly major proliferative cell components in ESCC TME. Tumour-associated macrophages and Tregs exert tumour-promoting effects by inducing immune escape in the ESCC TME (19, 20). The high infiltration of tumour-infiltrating lymphocytes is closely related to the favourable prognosis and clinical response of ESCC (21–23).

We aimed to characterise the pattern of immune cell infiltration (ICI) in ESCC. The CIBERSORT and single-sample gene set enrichment analysis (ssGSEA) algorithm was used to describe the ICI level of each sample. The ESTIMATE algorithm was applied to estimate immune and stromal scores. We further established ICI scores to predict the survival of ESCC patients.

## Materials and Methods

### ESCC Datasets and Pre-Processing

ESCC gene expression data was downloaded from The Cancer Genome Atlas (TCGA, https://portal.gdc.cancer.gov, TCGA-ESCC) and the Gene Expression Omnibus (GEO, https://www.ncbi.nlm.nih.gov/geo, GenBank: GSE53625). The patients without complete clinical information were excluded. The data of 206 patients were available for further analysis. The raw Fragments Per Kilobase per Million (FPKM) data from TCGA were converted into Transcripts Per Kilobase Million (TPM) data, which were similar to those of the GEO database to facilitate analysis. The transcriptomic data of ESCC from the GEO (GenBank: GSE53625) were annotated and normalised. The ComBat algorithm was used to remove batch effects due to non-biotech bias. Principal component analysis (PCA) analysis was performed to show the difference of two datasets before and after integration (**Figures S1A, B**). The detailed clinicopathological information of the ESCC samples are shown in **Table S1**.

### Consensus Clustering of ICI

We quantified the ICI level of each ESCC sample using the CIBERSORT and ssGSEA algorithm (24, 25). Previously reported immune-related gene signatures (26–28) were used to characterise different immune states. The immune and stromal scores of the ESCC samples were calculated using the ESTIMATE algorithm. Unsupervised clustering was performed by applying the ‘ConsensuClusterPlus’ package of R and iterated 1,000 times.

### Generation and Enrichment Analysis of ICI Signature Genes

The ESCC samples were divided into three ICI clusters based on ICI level, and ICI-related DEGs (|logFC| > 1.86, *P* < 0.05) were identified using the ‘limma’ package of R. Two gene clusters were identified according to ICI-related DEGs using the unsupervised clustering method. The ICI-related DEGs that were positively or negatively correlated with the gene cluster were defined as ICI signature genes A (ICISGA) or B (ICISGB). The ‘clusterProfiler’ package of R was used for Gene Ontology (GO) enrichment analysis, and the threshold was set as *P* < 0.05.

### Establishment of TME Scores

Boruta algorithm was used to reduce the dimensions of ICISGA and ICISGB, and PCA was used to extract the first principal component (PC1), which are the signature scores of ICISGA and ICISGB (defined as PC1A and PC1B, respectively). Finally, we constructed the ICI scores using the formula below. The ESCC samples were divided into the high and low ICI score groups according to the optimal threshold of ICI score. GSEA was performed and visualised using R package ‘ggplot2’.


ICI score=∑PC1A–∑PC1B

### Analysis of Tumour Mutation Profiles

ESCC mutation data were obtained from TCGA. The raw data were annotated with somatic Mutation Annotation Format, and the mutation signatures of the ESCC samples were characterised. The driver genes of each sample were identified by the ‘maftool’ package of R, and somatic alterations in the driver genes between the two ICI score groups were evaluated.

### Construction of Prognostic Model

Prognostic nomogram model was constructed to quantitatively predict the 1‐, 3‐ and 5‐year probability of death (PD), which consisted of the ICI score and clinical variables. Receiver operating characteristic (ROC) and calibration curves were plotted to indicate the discrimination ability of the prognostic model.

### Statistical Analyses

R (v3.6.3) or GraphPad Prism (v6.0) software were used for statistical analysis. Student’s t-test was used to compare the normally distributed variables between the two groups. Wilcoxon test was used to compare non-normally distributed variables between the two groups. The Kruskal–Wallis test was used to compare variables amongst the three groups. Kaplan–Meier method was used to generate the survival curve. The log-rank test was used to compare the differences. The optimal cut-off value of the data was evaluated with R’s ‘Survminer’ package, and the heatmap was generated by the ‘pheatmap’ package of R. Spearman correlation was applied to analyse the correlation between ICI score and TMB. P < 0.05 was considered statistically significant.

## Results

### ICI Profile in ESCC TME

**Figure 1:**
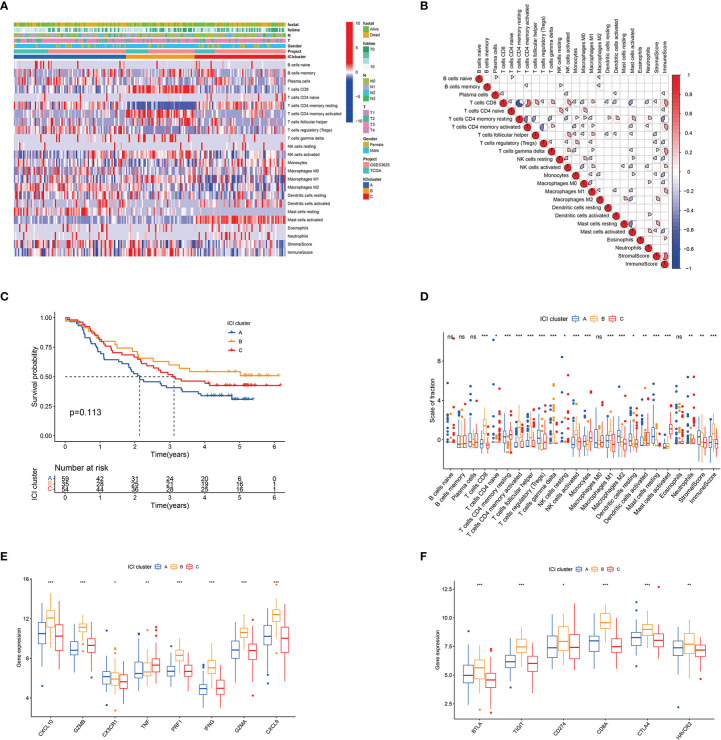
Landscape of ICI in the TME of ESCC. **(A)** Unsupervised clustering of infiltrating immune cells in ESCC samples. Rows denote infiltrating immune cells, and columns denote samples. **(B)** Heatmap of the intrinsic interaction of infiltrating immune cells, immune score and stromal score in TME. The colour from blue to red represents negative and positive correlations. The pie chart size represents the absolute correlation coefficient. **(C)** Kaplan–Meier curves for patients with ESCC in ICI clusters A–C. Log rank test *P* = 0.113. **(D)** Box plot of infiltration immune cell fractions in ICI clusters A–C. The immune and stromal scores of the three ICI clusters are also plotted. **(E)** Box plot of the expression levels of immune activity-related signature genes (*CXCL10*, *GZMB*, *CX3CR1*, *TNF*, *PRF1*, *IFNG*, *GZMA* and *CXCL9*) among ICI clusters A–C. **(F)** Box plot of the expression levels of immune checkpoint signature genes (*BTLA*, *TIGIT*, *CD274*, *CD8A*, *CTLA4* and *HAVCR2*) in ICI clusters A−C. *P < 0.05; **P < 0.01; ***P < 0.001; ns, no significance.

A total of 206 ESCC samples from TCGA-ESCC (n = 97) and GEO (GSE53625, n = 179) databases were analysed. The overall flow chart of the study is shown in **Figure S1C**. **Tables S2 , S3** list the ICI level, immune scores and stromal scores of the ESCC specimen obtained by CIBERSORT and ssGSEA algorithms, respectively. Unsupervised clustering was used to perform ICI clustering, with K = 3 (ICI clusters A–C) as the optimal cluster pattern (**Figures S1D–H**). The heatmap shows the differences in the composition of immune cells amongst ICI clusters A–C (**Figure 1A**). **Figure 1B** presents the pattern of immune cell interaction in ESCC TME. Survival analysis showed that ICI cluster A tended to have a worse survival rate than ICI clusters B and C, although the difference had no statistical significance (**Figure 1C**). Furthermore, ICI cluster A was marked by a low immune score with high densities of Tregs, M2 macrophages, resting mast cells and resting dendritic cells. ICI cluster B exhibited high levels of CD8 T cells, CD4 T cells, follicular helper T cells, activated NK cells and M1 macrophages. ICI cluster C was characterised by minimum immune and stromal scores and low densities of most immune cells, except for the resting memory CD4 T cells, activated mast cells and monocytes (**Figure 1D**). The expression levels of immune activity-related signature genes (*CXCL10*, *GZMB*, *PRF1*, *IFNG*, *GZMA* and *CXCL9*) and immune checkpoint signature genes (*BTLA*, *TIGIT*, *CD274*, *CD8A*, *CTLA4* and *HAVCR2*) were higher in ICI cluster B than in ICI clusters A and C, as shown in **Figures 1E, F**.

### Identification of Immune Gene Subtype

**Figure 2:**
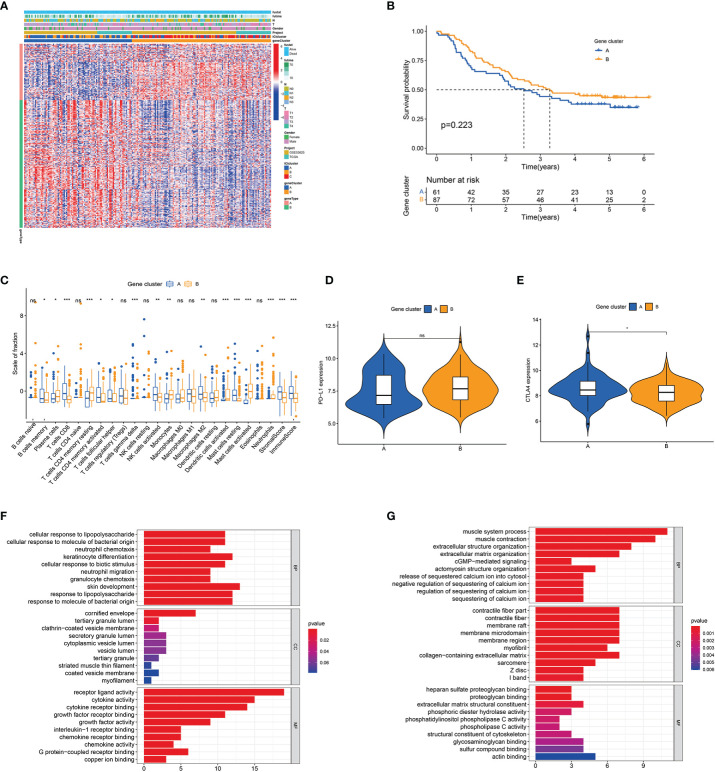
Identification of immune gene subtypes. **(A)** Unsupervised clustering of DEGs among three ICI clusters in ESCC cohorts. **(B)** Kaplan–Meier curves for patients in gene clusters A and **(B)** Log-rank test *P* = 0.040. **(C)** Box plot of infiltrating immune cell fractions in gene clusters A and **(B)** The immune and stromal scores of the two gene clusters are also plotted. **(D, E)** Expression levels of *PD-L1* **(D)** and *CTLA4* **(E)** between the two gene clusters. **(F, G)** GO enrichment analyses of ICISGA **(F)** and ICISGB **(G)**. The X-axis represents the number of genes within each GO term. *P < 0.05; **P < 0.01; ***P < 0.001; ns, no significance.

A total of 333 ICI-related differentially expressed genes (DEGs) amongst the three ICI clusters were identified by R’s ‘limma’ package (**Table S4**). Unsupervised cluster analysis was conducted, and two gene clusters (gene clusters A and B) had the optimal patterns (**Figures S2A–D**). **Figure 2A** shows the transcriptomic profiles of the 333 ICI-related DEGs between the two genomic clusters. Gene cluster A tended to have poorer outcome than gene cluster B but without statistical difference (**Figure 2B**). Gene cluster A had a massive infiltration of CD8 T cells, activated memory CD4 T cells, M2 macrophages, memory B cells, gamma delta T cells, resting mast cells, activated NK cells and follicular helper T cells. Alternately, gene cluster B contained a large number of plasma cells, monocytes, activated dendritic cells, activated mast cells, resting memory CD4 T cells and neutrophils (**Figure 2C**). Gene cluster A had relatively higher levels of immune checkpoint-related genes (*BTLA*, *TIGIT*, *CD8A* and *CTLA4*) than gene cluster B (**Figures 2D, E** and **S2F**). ICI signature genes A (ICISGA) and B (ICISGB) are shown in **Table S5**. The GO enrichment result revealed that ICISGA was involved in inflammation, whereas ICISGB was remarkably enriched in immune-related signalling pathways, such as T cell activation and B cell-mediated immunity (**Figures 2F, G** and **Table S6**).

### Construction of ICI Score

**Figure 3:**
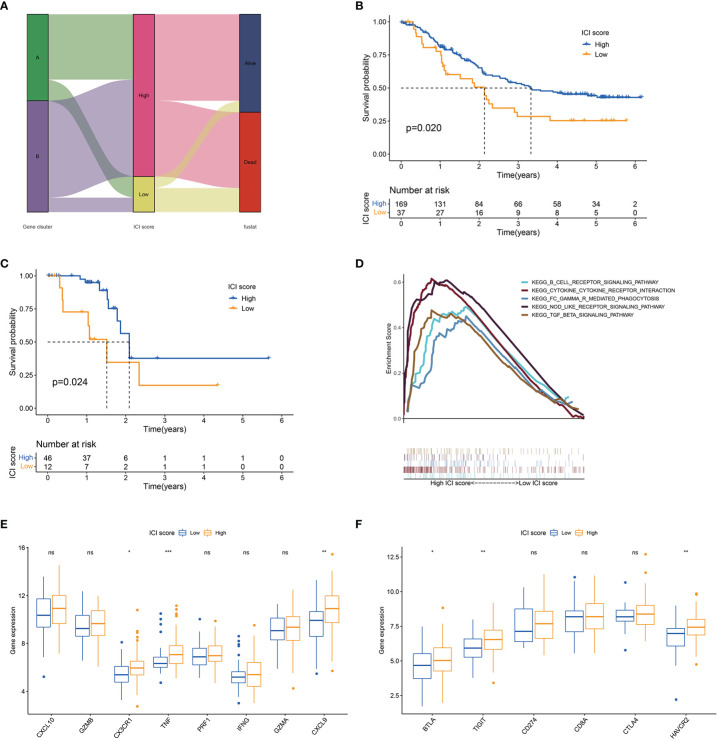
ICI score analysis. **(A)** Alluvial diagram of gene cluster distribution and survival outcome between high and low ICI score groups. **(B)** Kaplan–Meier curves for high and low ICI score groups in all samples. Log rank test *P* = 0.020. **(C)** Kaplan–Meier curves for high and low ICI score groups in the TCGA cohort. Log rank test *P* = 0.024. **(D)** GSEA of high and low ICI score groups. **(E)** Box plot of the expression levels of immune activity-related signature genes (*CXCL10*, *GZMB*, *CX3CR1*, *TNF*, *PRF1*, *IFNG*, *GZMA* and *CXCL9*) between high and low ICI score groups. **(F)** Box plot of the expression levels of immune checkpoint signature genes (*BTLA*, *TIGIT*, *CD274*, *CD8A*, *CTLA4* and *HAVCR2*) between high and low ICI score groups. *P < 0.05; **P < 0.01; ***P < 0.001; ns, no significance.

The abovementioned results suggested that ICI or gene cluster alone cannot accurately evaluate the prognosis of patients with ESCC. Two aggregate scores were calculated by principal component analysis (PCA), namely, (1) PC1A from ICISGA and (2) PC1B from ICISGB, to establish accurate prediction indicator for ESCC. ICI score was calculated from PC1A and PC1B based on the relevant scoring formula. Patients were classified into the high and low ICI score groups according to optimal score threshold (**Table S7**). As shown in **Figures 3A–C**, the high ICI score group had better survival rate than the low ICI score group. Accordingly, GSEA revealed that the B cell receptor, NOD-like receptor, Fc gamma R-mediated phagocytosis and immune suppression TGF-β signalling pathways were obviously enriched in the high ICI score group (**Figure 3D**). We observed that immune activity-related signature genes (*CX3CR1*, *TNF* and *CXCL9*) and immune checkpoint signature genes (*BTLA*, *TIGIT* and *HAVCR2*) were significantly higher in the high ICI score group than in the low ICI score group (**Figures 3E, F**).

### ICI Score and Tumour Mutation Burden (TMB)

**Figure 4:**
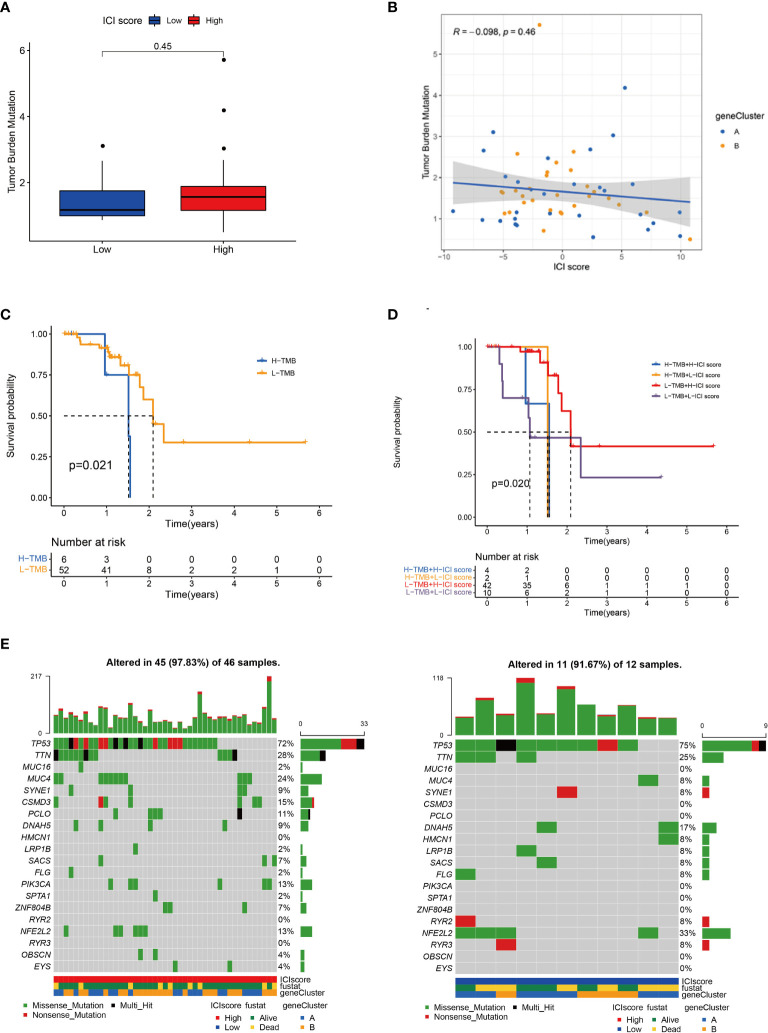
Correlation between ICI score and TMB. **(A)** TMB difference between high and low ICI score groups. **(B)** Scatter plots of the Spearman correlation between ICI score and TMB. **(C)** Kaplan–Meier curves for high and low TMB groups. Log rank test *P* = 0.021. **(D)** Kaplan–Meier curves for patients stratified by TMB and ICI score. Log rank test *P* = 0.020. **(E)** OncoPrint of high (left) and low (right) ICI score groups. Each column represents a single patient. The bar chart at the top represents TMB. The bar chart on the right shows the mutation frequency of each gene in the group with high or low ICI score.

A tumour with high mutation frequency may act as an effective biomarker for the efficacy of immunotherapy (29). In the present study, we evaluated the difference in mutation frequencies between the high and low ICI score groups. No remarkable difference was found in the mutation frequency between the two groups (**Figure 4A** and **Table S8**). Tumour mutation burden (TMB) and ICI score had no statistical correlation (**Figure 4B**). In addition, we concluded that a patient with high TMB had worse outcome than that with low TMB (**Figure 4C**). Stratified analysis showed that in the low TMB subgroup, the patients with high ICI score had a remarkable survival advantage compared with the patients with low ICI score, whereas no statistical difference was found between the two ICI score groups in the high TMB subgroup (**Figure 4D**). The oncoPrint presented the top 20 high-frequency mutated genes (**Figure 4E**). The ICI score could predict survival independent of TMB.

### ICI Score and Clinicopathological Features

**Figure 5:**
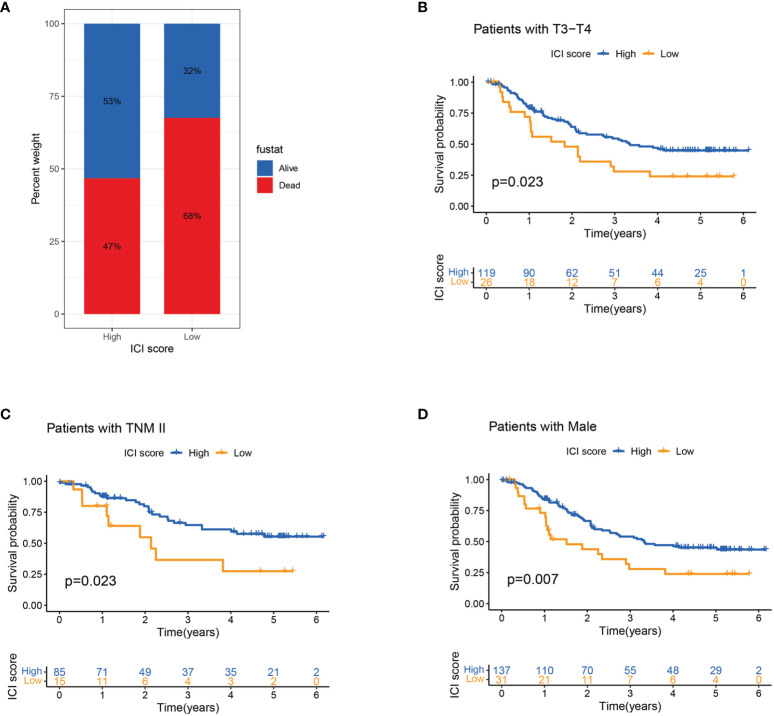
Association of ICI score and clinicopathological features. **(A)** Survival rates of patients with ESCC in high and low ICI score groups. **(B–D)** Kaplan–Meier curves for high and low ICI score groups in patients with T3–T4 stage **(B)** and TNM II stage **(C)** and in male patients **(D)** in the entire cohort.

Age, gender, depth of tumour invasion and pathological stage were related to the prognosis. This study evaluated the predictive ability of ICI score in the stratification of different clinicopathological features. Compared with the low ICI score group, the high ICI score group had more surviving patients (**Figure 5A**). Stratification analysis revealed that patients with high ICI score had superior survival than patients with a low ICI score in the T3–T4, TNM II and male patient groups (**Figures 5B–D**). ICI score may be a predictor of survival in T3-T4, TNM II and male patients’ groups.

### ICI Score and Prognosis

**Figure 6:**
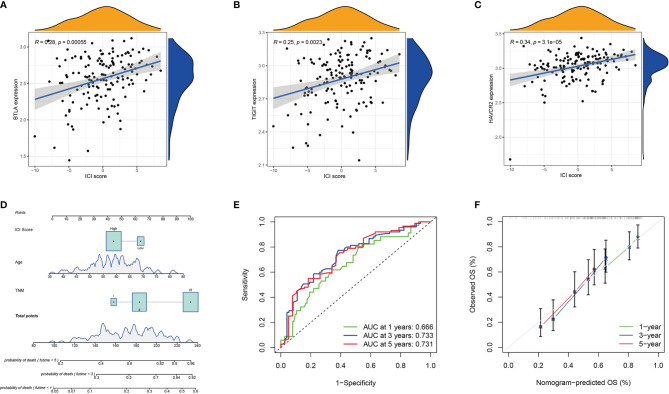
Prognosis Significance of ICI Scores. **(A–C)** Correlation analysis between the ICI score with the three differentially expressed immune checkpoint signature genes (*BTLA*, *TIGIT*, and *HAVCR2*). *BTLA* **(A)**, *TIGIT* **(B)**, and *HAVCR2* **(C)**; **(D)** The nomogram for predicting 1-, 3-, or 5- year probability of death; **(E)** The ROC curve of the nomogram for predicting the 1-, 3-, or 5- year probability of death. **(F)** The calibration curve of the nomogram for predicting the 1-, 3-, or 5- year probability of death.

To evaluate the potential immune activity of ICI score in ESCC. The correlation between the ICI score and the three differentially expressed immune checkpoint signature genes (*BTLA*, *TIGIT* and *HAVCR2*) were analysed (**Figures 6A–C**). The ICI score was positively correlated with BTLA (R=0.28, *P*<0.001), TIGIT (R=0.25, *P*=0.0023) and HAVCR2 (R=0.34, *P*=0.0023), suggesting that ICI score may play a non-negligible role in predicting the response of ESCC patients to immune checkpoint inhibitors treatment. In addition, nomogram was constructed to predict 1-, 3- and 5-year PD (**Figure 6D**). The ROC and calibration curves indicated that the model has a good discrimination ability (AUC were 0.666 for a 1-year PD, 0.733 for a 3-year PD and 0.731 for 5-year PD; **Figures 6E, F**).

## Discussion

The advent of immunotherapy has rapidly changed the treatment paradigm for multiple cancers (30, 31). Currently, only anti‐PD‐1/PD-L1 immunotherapy drugs have been approved by the FDA for ESCC treatment (32). Pembrolizumab was approved as the second-line drug for PD-L1-positive advanced ESCC in 2019 (11). Nivolumab was approved in 2020 for patients with unresectable ESCC who previously underwent chemotherapy with fluorouracil and platinum regardless of PD-L1 expression level (33). Although immunotherapy has shown remarkable clinical efficacy in a variety of malignant tumours, only a small number of patients benefit from it (34, 35), which underscores the importance of identifying suitable patients. We comprehensively delineated the immune landscape of ESCC and constructed ICI score to predict patient survival outcomes.

Previous evidence revealed that tumour-specific immune cell dysfunction contributes to immune evasion, leading to tumour survival and progression (36, 37). In the present study, we firstly divided the ESCC samples into three ICI clusters by unsupervised clustering. ICI cluster A presented immunosuppressive phenotype with high stromal and low immune scores, and this was accompanied by high infiltration of Tregs and M2 macrophages. ICI cluster B presented an immunoactivated phenotype with high immune and low stromal scores, and this was accompanied by high levels of CD8 T cells, activated memory CD4 T cells and activated NK cells. ICI cluster C showed the lowest immune and stromal scores and exhibited an immune desert phenotype. Then, we analysed ICI-related DEGs and defined two gene clusters. Compared with gene cluster B, gene cluster A had higher densities of CD8 T cells, memory B cells, CD4 T cells, M2 macrophages and activated NK cells. Although gene cluster A presented an immune-activated phenotype, it is marked by high levels of BTLA, TIGIT, CD8A and CTLA-4. Compared with gene cluster B, the prognosis of gene cluster A tended to be poorer, but no remarkable difference was found. The above result highlighted the fact that ICI had an anti-tumour effect but cannot accurately predict the survival outcome of patients.

Given the complex tumour heterogeneity, we used Boruta algorithm to construct ICI scores to provide a more comprehensive classification scheme. GSEA revealed that the high ICI score group was associated with immune activity-related pathway, including B cell receptor, Fc gamma R-mediated phagocytosis, NOD-like receptor and immune suppression TGF-β signalling pathway (38, 39). The low ICI score group exhibited relatively low immune activities, thereby implying an immune cold phenotype. Compared with the patients in the low ICI score group, the patients in the high ICI score group had better prognoses. A large number of studies have shown that somatic mutations are likely to give rise to high neoantigen levels (40–42) and therefore could attract immune cells involved in anti-tumour immune response (43, 44). We downloaded and analysed ESCC mutation data to exclude the influence of TMB on the predictive ability of ICI score. High TMB led to poor prognosis in ESCC, which was similar to the findings of previous studies (45, 46). However, no correlation was found between ICI score and TMB in ESCC. The ICI score may be a prognostic predictor for ESCC that is independent of TMB.

Immune checkpoints are a class of immunosuppressive molecules expressed on immune cells and that regulate the degree of immune activation; these include *PD1*, *CTLA4*, *TIGIT*, *BTLA* and so on (47). In addition to the well-known immune checkpoint PD1, TIGIT has also been reported as a potential target for the treatment of malignant tumors (48, 49). In January 2021, tiragolumab (anti-TIGIT) combined with atezolizumab was approved by FDA for the treatment of metastatic non-small cell lung cancer patients with PD-L1 but without EGFR/ALK genome abnormalities. The role of other immune checkpoints in cancer immunotherapy is being studied. In this study, compared with low ICI score group, the three immune checkpoint-related genes (*BTLA*, *TIGIT* and *HAVCR2*) in the high ICI score group were highly expressed. The correlation analysis revealed that the three immune checkpoint-related genes were positively correlated with ICI score. *BTLA*, *TIGIT* and *HAVCR* may serve as new potential therapeutic targets that possibly bring clinical benefits to ESCC patients with high ICI score. The ICI score may play a non-negligible role in predicting the response of ESCC patients to immune checkpoint inhibitor treatment.

Wang et al. revealed that high expressions of PD-L1, TIM3 and TIGIT were associated with poor prognosis of ESCC patients, and they constructed a nomogram model composed of PD-L1, TIM3, TIGIT and TNM stages to predict the prognosis of patients (50). Their data analysis involved only one ESCC dataset downloaded from TCGA, and the nomogram model involved only a few immune checkpoint-related molecules. Given the complex tumour heterogeneity, we established a prognostic nomogram model that included ICI score, age and TNM staging to predict patient survival. The ROC and calibration curves indicated that the nomogram model had good discrimination ability.

We depicted a comprehensive ICI pattern of ESCC and constructed ICI scores, which facilitated the understanding of the TME of ESCC and provided new prognostic biomarkers and potential targets for immunotherapy. However, this study had a limitation. None of the patients in the analysis received immunotherapy. Thus, the predictive value of ICI score for immunotherapy efficacy in patients with ESCC cannot be evaluated. Further studies will be carried out in the future.

## Data Availability Statement

The datasets presented in this study can be found in online repositories. The names of the repository/repositories and accession number(s) can be found in the article/**Supplementary Material**.

## Author Contributions

ZY a n d J Z conceived and designed the whole project; Z S, X W and LD analyzed study data and wrote the manuscript; HW and LY acquired and analyzed study data. All authors contributed to the article and approved the submitted version

## Funding

This work was supported by Shenzhen Key Medical Discipline Construction Fund (No. SZXK075) and Sanming Project of Medicine in Shenzhen (No. SZSM201612097), and Shenzhen Key Laboratory of Metabolic Health (No. ZDSYS20210427 152400001).

## Conflict of Interest

The authors declare that the research was conducted in the absence of any commercial or financial relationships that could be construed as a potential conflict of interest.

## Publisher’s Note

All claims expressed in this article are solely those of the authors and do not necessarily represent those of their affiliated organizations, or those of the publisher, the editors and the reviewers. Any product that may be evaluated in this article, or claim that may be made by its manufacturer, is not guaranteed or endorsed by the publisher.
