# Supplementary material for: Characterization of the Immune Cell Infiltration Landscape in Esophageal Squamous Cell Carcinoma
Source: Front Oncol. 2022 Jul 7;12:879326. doi: 10.3389/fonc.2022.879326 (PMC9300817; doi:10.3389/fonc.2022.879326)
Supplement: Supplementary file 1 [file DataSheet_1.docx]

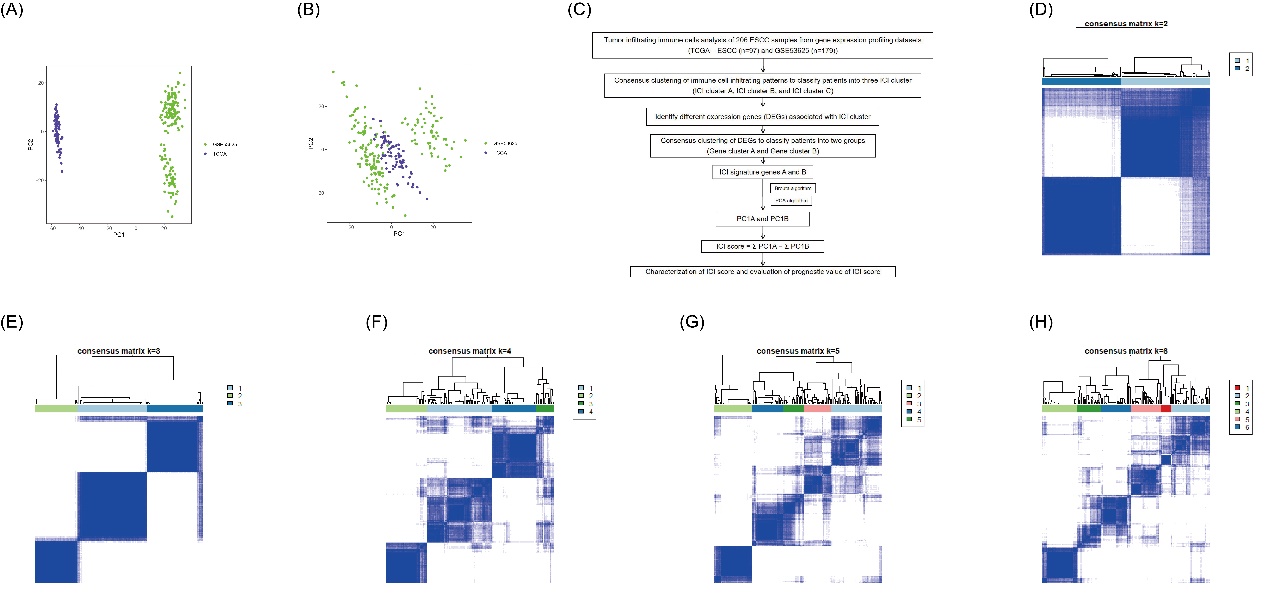


**Figure S1**. **(A and B)** The difference of two datasets before **(A)** and after **(B)** integration. **(C)**Flow chart of study design. **(D–H)** Consensus matrixes (K = 2–6) of ICI cluster for all ESCC samples.


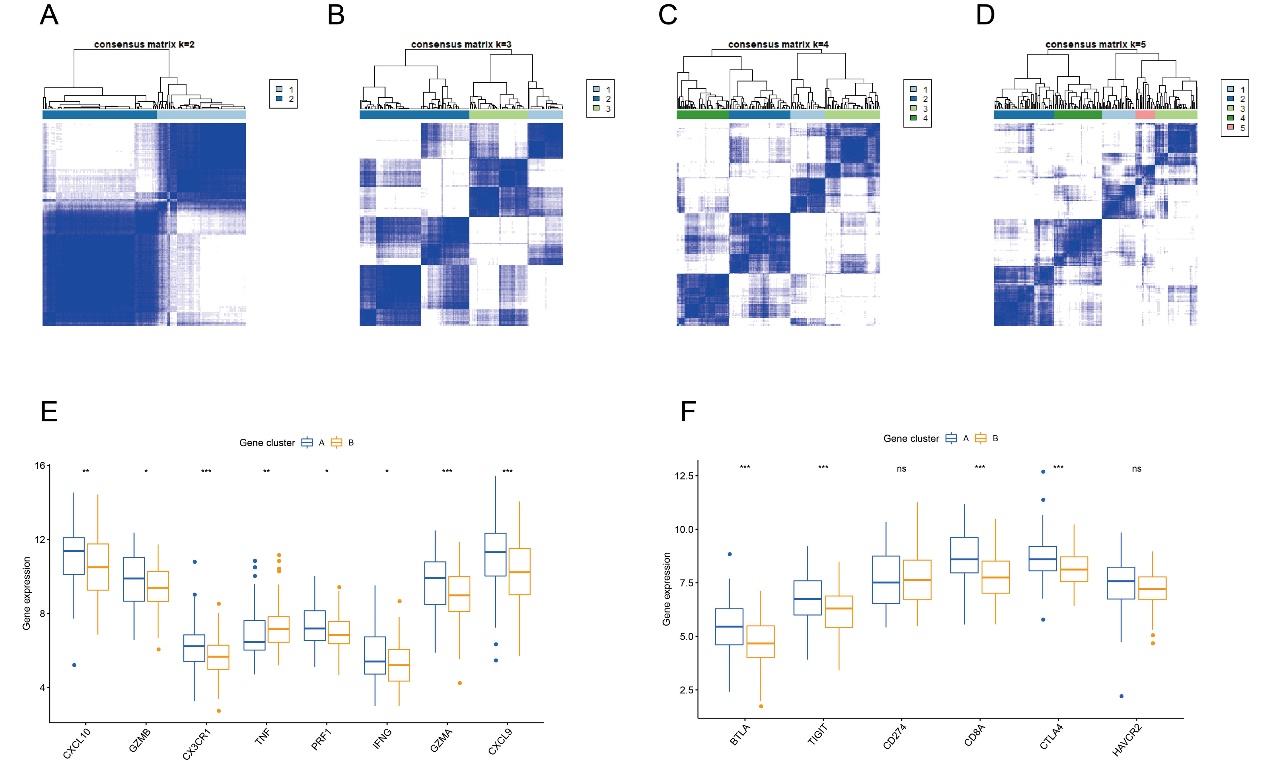


**Figure S2**. **(A–D)** Consensus matrixes (K = 2–5) of gene clusters for all ESCC sample. **(E)** Box plot of the expression levels of immune activity-related signature genes (*CXCL10*, *GZMB*, *CX3CR1*, *TNF*, *PRF1*, *IFNG*, *GZMA* and *CXCL9*) between gene clusters A and B. **(F)** Box plot of the expression levels of immune checkpoint signature genes (*BTLA*, *TIGIT*, *CD274*, *CD8A*, *CTLA4* and *HAVCR2*) between gene clusters A and B. * *P* < 0.05; ** *P* < 0.01; *** *P* < 0.001; ns: no significance.
